# Vitamin D Attenuates Oxidative Damage and Inflammation in Retinal Pigment Epithelial Cells

**DOI:** 10.3390/antiox8090341

**Published:** 2019-08-24

**Authors:** Ali Mohammad Tohari, Reem Hasaballah Alhasani, Lincoln Biswas, Sarita Rani Patnaik, James Reilly, Zhihong Zeng, Xinhua Shu

**Affiliations:** 1 Department of Clinical Biochemistry, King Fahad Hospital, PO Box 204, Jazan 91991, Saudi Arabia; 2 Department of Biological and Biomedical Sciences, Glasgow Caledonian University, Glasgow G4 0BA, UK; 3 Department of Bioengineering and Environmental Science, Changsha University, Changsha 410022, China; 4 Department of Vision Science, Glasgow Caledonian University, Glasgow G4 0BA, UK

**Keywords:** vitamin D, oxidative stress, inflammation, retinal pigment epithelial cells, age-related macular degeneration

## Abstract

Age-related macular degeneration (AMD), the most common visual disorder in elderly people, is characterized by the formation of deposits beneath the retinal pigment epithelium (RPE) and by dysfunction of RPE and photoreceptor cells. The biologically active form of vitamin D, 1,25-(OH)2D3 (VITD), is categorized as a multifunctional steroid hormone that modulates many transcriptional processes of different genes and is involved in a broad range of cellular functions. Epidemiological and genetic association studies demonstrate that VITD may have a protective role in AMD, while single nucleotide polymorphisms in the vitamin D metabolism gene (*CYP24A1*) increase the risk of AMD. However, the functional mechanisms of VITD in AMD are not fully understood. In the current study, we investigated the impact of VITD on H_2_O_2_-induced oxidative stress and inflammation in human RPE cells. We demonstrate that exposure to H_2_O_2_ caused significantly reduced cell viability, increased production of reactive oxygen species (ROS), lowered expression of antioxidant enzymes and enhanced inflammation. VITD exposure notably counteracted the above H_2_O_2_-induced effects. Our data suggest that VITD protects the RPE from oxidative damage and elucidate molecular mechanisms of VITD deficiency in the development of AMD.

## 1. Introduction

Age-related macular degeneration (AMD) is an accelerating neurodegenerative ocular disease that affects people above 55 years of age [1]. Epidemiologic studies have shown that there is an increasing number of new AMD cases diagnosed worldwide annually [2]. Wong et al. (2014) reported that the incidence of AMD is 7.1% in the African, 6.81% in the Asian, 11.19% in the Caucasian and 9.87% in the Hispanic population [3]. Several risk factors, such as smoking, have been associated with increased incidence of AMD [2]. A number of studies have suggested that the incidence of AMD is also linked to genetic defects [1,2]. It has been proposed that the AMD genetic association is strongly related to genetic defects in ARMS2/HTRA1 and CFH located on, respectively, chromosome 10q26 and 1q32 [1,2]. Although the pathological mechanisms of AMD are not fully understood, many studies have suggested that AMD is an inflammatory process that involves increased production of many inflammatory mediators such as tumor necrosis factor-α (TNF-α), interleukins-1β, 6, 8 and 33 (IL-1β, IL-6, IL-8 and IL-33) [1,4], vascular endothelial growth factor A (VEGFA) [5] and activation of the alternative complement pathway [6].

Vitamin D is a fat-soluble secosteroid molecule that is found in two forms: vitamin D_2_ and vitamin D_3_ [7]. The latter is more abundant than vitamin D_2_ in biological systems; thus, vitamin D_3_ is commonly referred to simply as vitamin D. Humans and higher mammals can obtain vitamin D from various nutritional sources such as meats and fungi [7]. In addition, vitamin D can be produced endogenously. The endogenous synthesis of vitamin D starts in the epidermal skin layers where the vitamin D precursor 7-dehydrocholesterol (7-DHC) is converted to pre-vitamin D_3_ through a photoisomerization process activated by exposure to type B ultraviolet radiation (UVB) between 280 and 320 nm, then incorporated with its carrier globin, vitamin D binding protein (DBP), and transported to the liver [7]. The pre-vitamin D_3_ is transformed to 25-dihydroxyvitamin D_3_ through the hepatic cytochrome P450 hydroxylase enzymes microsomal CYP2R1 and mitochondrial CYP2A1, attached to DBP and transported to the kidney [7]. Finally, 25-dihydroxyvitamin D_3_ is converted to the active form 1,α,25-dihydroxyvitamin D_3_ (1,25(OH)_2_D_3_) in renal cells by mitochondrial 1,α, CYP27B1. This step is tightly regulated by parathyroid hormone (PTH) and by circulating levels of calcium and phosphate, as well as by 1,25(OH)_2_D_3_ itself [7]. The molecule 1,α,25-dihydroxyvitamin D_3_ is well recognized as a steroid hormone that regulates many cellular signalling activities through its nuclear vitamin D receptor (VDR) in target cells [7,8]. Vitamin D deficiency in the general population is currently considered a global health concern and represents a risk factor for the development of many diseases, particularly neurodegenerative diseases [9]. A large number of studies have shown that the likelihood of AMD is increased among those individuals with low circulating vitamin D levels [10,11,12,13].

IL-33 is a member of the IL-1 cytokine family that is expressed in many cell types of the nervous system [14]. IL-33 signalling is mediated through binding to its ST2 receptor in target cells and recruiting transcriptional cofactors such as IL-1R accessory protein (IL-1RAP), IL-1R-associated kinases 1/4 (IRAK1/4) and myeloid differentiation primary-response protein 88 (MYD88) into the transcription complex [14,15]. This cytokine has important roles in the inflammatory response in the modulation of neurodegenerative diseases, e.g., Alzheimer’s disease [16] and AMD [4].

In the current study, we investigated the role of vitamin D in oxidative stress and inflammation. We found that vitamin D increased cell viability, reduced the formation of reactive oxygen species (ROS), and inhibited inflammation. Vitamin D also stimulated the expression of IL-33 at the mRNA and protein levels. Furthermore, IL-33 treatment markedly induced the expression of antioxidant genes. Our data suggest that the protective effects of vitamin D in AMD may be partially through the upregulation of IL-33.

## 2. Materials and Methods

### 2.1. Cell Culture

ARPE-19 cells (ATCC^®^ CRL-2302^™^, ATCC, Manassas, VA, USA) were grown in a T-25 cm^2^ standard tissue culture flask in 5 mL of DMEM/F12 medium containing 2.4 mM L-Glutamine, 15 mM Hepes, 17.5 mM glucose, 10% FBS, 50 IU/mL of Penicillin and Streptomycin and 17.4 mL of 7.5% Sodium bicarbonate. The cells were maintained in a 5% CO_2_ incubator at 37 °C. Once cell growth reached 80–90% confluency, the medium was removed and the cells were washed twice with PBS. The cells were then trypsinized with 1–1.5 mL of 0.5% trypsin–EDTA. Detached cells were resuspended in 4–5 mL of DMEM/F12 medium and seeded in 96- or 6-well tissue culture plates for 24 h at a density of 50,000 or 500,000 cells per well prior to cell treatment.

### 2.2. Cell Viability Assay

ARPE-19 cells were seeded at a density of 50,000 cells per well in a 96-well plate in DMEM/F12 medium for 24 h. The medium was then removed, and the cells were washed twice with 0.5 mL of 1× PBS/well. The cells were then treated with serial concentrations of H_2_O_2_ (150–2000 µM), 50 nM 1α, 25(OH)_2_D_3_ (VITD), 0.1% ethanol for 6 and 24 h (or left untreated for the same time periods). In some experiments, ARPE-19 cells were treated with 750 µM H_2_O_2_, 750 µM H_2_O_2_ + 50 nM VITD, or left untreated. All treatments were performed in serum-free DMEM/F12 medium. The cell viability assay was performed as previously described [17].

### 2.3. Caspase-3/7 Activity

ARPE-19 cells were seeded in a 96-well plate (20,000 cells/well) overnight and treated with 750 µM H_2_O_2_, 750 µM H_2_O_2_ + 50 nM VITD, or 0.1% DMSO (control). Caspase-3/7 activity was measured by the Apo-ONE Homogeneous Caspase-3/7 kit (Promega, Southampton, UK) according to the manufacturer’s instructions. Fluorescence was measured using a Fluostar Optima Plate Reader at an excitation wavelength of 499 nm and an emission wavelength of 521 nm. Caspase-3/7 activity was determined according to the following formula: % relative fluorescence = (fluorescence of treated cells − fluorescence of control cells)/fluorescence of control cells × 100. All experiments were repeated three times.

### 2.4. Measurement of Reactive Oxygen Species (ROS)

ARPE-19 cells were cultured at a density of 25,000 cells per well in a 96-well plate and treated with 750 µM H_2_O_2_, 750 µM H_2_O_2_ + 50 nM VITD, or 0.1% DMSO for 6 and 24 h. Production of ROS was measured using the 6-Carboxy-2′,7′-Dichlorofluorescin diacetate (DCFH-DA) dye (Sigma, Dorset, UK). The medium was removed, and the cells were washed twice with 1× PBS, 200 μL/well. Then, 150 μL of 10 μM DCFH-DA in 1× PBS/well was added, and the plate was incubated for 45 min in a 5% CO_2_ incubator at 37 °C. Fluorescence was measured at 485 nm (excitation) and 525 nm (emission) using a Fluostar Optima microplate reader (BMG LABTECH Ltd, Bucks, UK). The ROS level was represented as the percentage of fluorescence intensity relative to the control after baseline fluorescence correction according to the following formula: (fluorescence of treated cells − fluorescence of control cells)/fluorescence of control cells × 100. All experiments were repeated three times.

### 2.5. Enzyme-Linked Immunosorbent Assay (ELISA)

Total proteins from ARPE-19 cells, retina and RPE were extracted by homogenizing samples in 200 μL of T-PER buffer containing 1× protease Inhibitor Cocktail. The supernatants were collected by centrifugation at 10,000 rpm for 5 min. The concentration of IL-1β, IL-8, IL-33, TNFα and VEGFA in control and treated human ARPE-19 cell media was measured using commercial ELISA kits from PeproTech and eBioscience according to the manufacturer’s protocols. The levels of the examined cytokines were quantified using a standard curve constructed on the basis of the mean absorbance of standard solution concentrations of cytokines (*X* axis) against optical density (O.D). for each standard solution (*Y* axis). The concentration of unknown samples (pg/mL) was calculated on the basis of the straight line equation obtained from the linear-regression trendline according to *Y* = *mx* + *c* (where *Y* = O.D. of unknown sample, *m* = slope value, *x* = concentration of unknown sample and *c* = intercept). All experiments were repeated three times.

### 2.6. Biochemical Assay

Quantification of malondialdehyde (MDA) and glutathione (GSH) and measurement of CATALASE and superoxide dismutase (SOD) activities were performed using kits from CELL BIOLABS INC (San Diego, CA, USA) according to the manufacturer’s instructions.

### 2.7. Mouse Samples

Mice used in this study were housed in the Animal Unit at Glasgow Caledonian University with free access to food and water and a 12 h light/dark environment. Mice at the age of 2 months, 9 months and 24 months were sacrificed, and the retina and RPE were dissected and stored at −80 °C for further analysis. Approval for animal use was granted by the Glasgow Caledonian University Animal Ethics and Welfare Committee, in accordance with the UK home office animal care guidelines (Project licence P8C815DC9).

### 2.8. Quantitative Real-Time Polymerase Chain Reaction (qRT-PCR)

Total RNAs from ARPE-19 cells and mouse tissues (retina and RPE) were extracted using Tri Reagent^®^ (Sigma, Dorset, UK) following the manufacturer’s instructions. The cDNA was synthesized using the High-Capacity cDNA Reverse Transcription kit (Thermo Fisher Scientific, Paisley, UK) as described by the manufacturer. Quantification of gene expression was performed using real-time PCR Platinum SYBR Green qPCR SuperMix-UDG with ROX assay, as described by the manufacturer. Briefly, 1.0 µL of 50 ng/µL of cDNA was mixed with 7.5 µL of Platinum SYBR Green qPCR SuperMix-UDG with ROX and 0.6 µl of 10 µM forward and reverse primers, and the reaction volume was scaled to 15 µL with nuclease-free water. DNA amplification was carried out under the following conditions: 50 °C for 2 min (UDG incubation), followed by enzyme activation at 95 °C for 2 min, hold and then an amplification step of 40 cycles including DNA denaturation at 95 °C for 15 s, then primer annealing at 60 °C for 15 s. Fluorescence signals were detected at the end of the 60 °C step, and assay validity was assessed on the basis of the melting curve analysis following each run. Relative gene expression was determined according to the 2^−ΔΔct^ formula. The primer sequences for qRT-PCR are available on request.

### 2.9. Immunostaining

ARPE-19 cells were fixed with methanol at −20 °C for 5 min, then washed with 1× PBS twice. The cells were blocked with 2% BSA-PBS at room temperature for 30 min, then incubated with primary antibodies at 4 °C overnight. After washing three times (5 min each time), the cells were blocked again with 2% sheep serum in 2% BSA-PBS for 30 min. The cells were incubated with secondary antibodies at room temperature for 1 h, then washed 5 times with 1× PBS (5 min each). The cells were mounted with DAPI solution and imaged under a confocal microscope.

### 2.10. Western Blot

Treated and control cells were lysed with Radio-Immuno Precipitation Assay (RIPA) buffer. Proteins were separated by SDS-PAGE and transferred to nitrocellulose membranes. The targeted proteins were detected using primary antibodies (NRF2, 1:1000; GAPDH, 1:1000) and secondary antibodies (1:10,000). The signals were quantified with the ImageStudio™Lite analysis software (LI-COR, Cambridge, UK).

### 2.11. Statistical Data Analysis

Data were analysed by one-way or two-way Anova followed by Bonferroni post-hoc test using GraphPad Prism version 6 software (GraphPad Software Inc., San Diego, CA, USA); *p* < 0.05 was considered significant. All experiments were repeated three times.

## 3. Results

### 3.1. VITD Treatment Improved Cell Viability and Reduced ROS Production and Apoptosis

**Figure 1:**
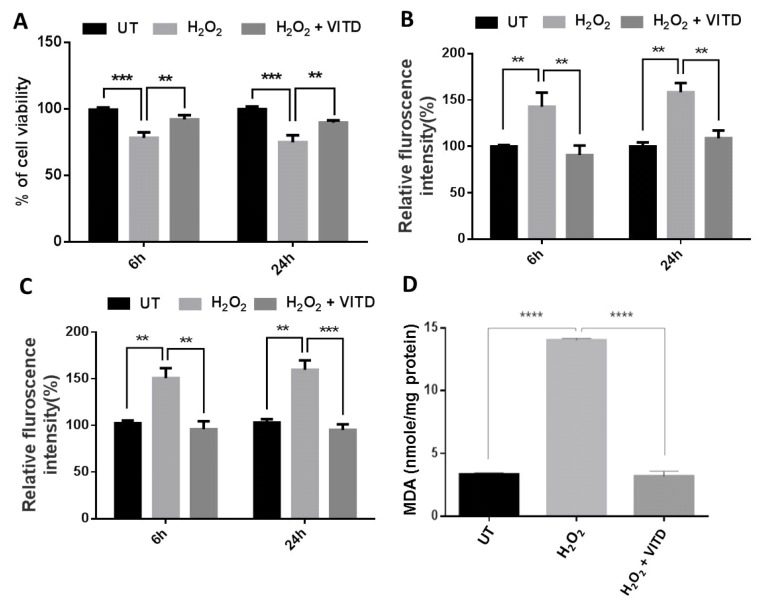
Effects of vitamin D (VITD, 1,25(OH)_2_D_3_) on cell viability, reactive oxygen species (ROS) production, apoptosis and malondialdehyde (MDA) level. ARPE-19 cells were treated with H_2_O_2_ alone or with H_2_O_2_ + VITD for 6 h or 24 h. Cell viability (**A**), ROS production (**B**), Caspase 3/7, a biomarker of apoptosis (**C**), and MDA, a biomarker of lipid peroxidation, levels (**D**) were examined. Data are presented as the means ± standard deviation (SD) of three independent experiments. UT: untreated control. ** *p* < 0.01, *** *p* < 0.001, **** *p* < 0.0001.

In order to determine the appropriate concentration of H_2_O_2_ to induce a significant, though not excessive, toxic effect on cell viability, ARPE-19 cells were challenged with H_2_O_2_ at a range of concentrations (150 to 2000 μM) for 6 or 24 h. We detected a significant reduction in cell viability in cells treated with 450, 600, 750 and 1000 μM H_2_O_2_ for 6 and 24 h compared to the respective control cells. A concentration of H_2_O_2_ higher than 1000 μM was determined to be highly toxic (Figure S1). Thus, 750 μM H_2_O_2_ was used to treat ARPE-19 cells for all subsequent experiments in this study. We also chose 50 nm VITD for our current study on the basis of earlier in vitro studies [18,19]. We found that 50 nM VITD significantly increased the viability of treated ARPE-19 cells when compared to untreated control cells (Figure S1). Previous studies reported that VITD treatment can protect cells and tissues from oxidative damage [20,21,22,23]. In the current study, treatment of ARPE-19 cells with 750 µM H_2_O_2_ for both 6 and 24 h caused a significant reduction in cell viability and a significant increase in ROS production (Figure 1A,B). The latter can activate apoptosis signalling pathways [24]. In the current study, caspase 3/7 activation, an indicator of apoptosis, was significantly increased in cells exposed to 750 µM H_2_O_2_ for both 6 and 24 h (Figure 1C). When H_2_O_2_ exposure was accompanied by a treatment with VITD, the above effects were reversed: there was a significant increase in cell viability, a significant decrease in ROS production and a significant decrease in caspase 3/7 activation, compared to the levels seen following treatment with H_2_O_2_ alone (Figure 1A–C). Increased ROS can promote lipid peroxidation, resulting in increased production of MDA and 4-hydroxynonental (4-HNE), which indicates local cell or tissue damage. We found that MDA production in ARPE-19 cells treated with H_2_O_2_ was significantly higher than that seen in untreated cells; co-treatment with VITD significantly counteracted this effect (Figure 1D).

### 3.2. VITD Treatment Enhanced VDR) Expression in Stressed ARPE-19 Cells

VDR is the principal nuclear receptor for VITD signalling in biological systems [7,8]. It is expressed in many cells including retinal and RPE cells [25,26,27]. Our current results show that VDR was expressed in untreated (control) ARPE-19 cells, in cells treated with H_2_O_2_ alone and in cells co-treated with H_2_O_2_ and VITD (Figure S2A). This expression was significantly reduced (upon both 6 and 24 h exposure) in ARPE-19 cells treated with 750 μM H_2_O_2_ compared to untreated cells; however, VDR expression was substantially increased (upon both 6 and 24 h exposure) in ARPE-19 cells co-treated with 750 μM H_2_O_2_ and 50 nM VITD compared to cells treated with 750 μM H_2_O_2_ alone (Figure S2B).

### 3.3. VITD Treatment Upregulated the Expression of Antioxidant Genes

**Figure 2:**
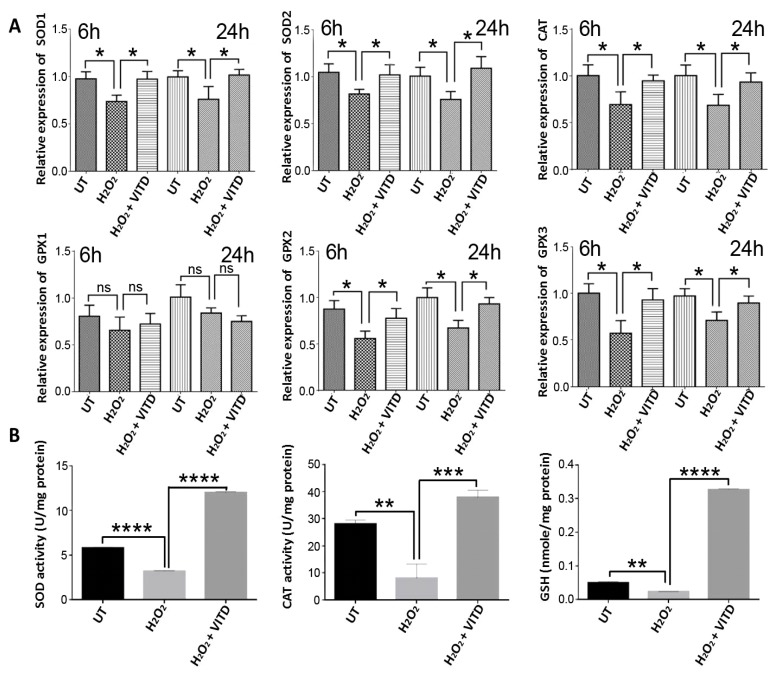
VITD treatment enhanced the expression of antioxidant genes. (**A**) ARPE-19 cells were exposed to H_2_O_2_ alone or H_2_O_2_ + VITD for 6 h or 24 h. The expression of *SOD1*, *SOD2*, *CATALASE* (CAT), *GPX1*, *GPX2* and *GPX3* in treated and untreated cells was measured by qRT-PCR. (**B**) ARPE-19 cells were exposed to H_2_O_2_ alone or H_2_O_2_ + VITD for 24 h; the activities of SOD and CAT and GSH level were biochemically examined. Data are presented as the means ± standard deviation (SD) of three independent experiments. UT: untreated control. ns: no significance. * *p* < 0.05, ** *p* < 0.01, *** *p* < 0.001, **** *p* < 0.0001.

Increased intracellular ROS production associated with many pathological conditions can cause major cellular injury, reducing cells’ capacity to produce protective molecules such as anti-oxidant proteins [28]. Recent studies have shown that VITD treatment can modulate both gene expression and activity of many antioxidant molecules [29,30,31]. In the current study, we used qRT-PCR to examine the expression of *SOD1*, *SOD2*, *CATALASE (CAT)*, *GPX1*, *GPX2* and *GPX3* genes in ARPE-19 cells under our stress condition and VITD treatment. We found that, at both 6 and 24 h, the expression of *CAT*, *SOD1*, *SOD2*, *GPX2* and *GPX3* were noticeably reduced in ARPE-19 cells treated with 750 μM H_2_O_2_ compared to untreated cells; however, the expression of these antioxidant genes was significantly increased in ARPE-19 cells co-treated with 750 μM H_2_O_2_ and 50 nM VITD compared to cells treated with 750 μM H_2_O_2_ alone (Figure 2A). There was no significant difference at 6 or 24 h in *GPX1* expression in untreated cells, cells treated with 750 μM H_2_O_2_ alone, and cells co-treated with 750 μM H_2_O_2_ and 50 nM VITD (Figure 2A).

We also examined the effect of VITD treatment on SOD and CAT activities. The activities of SOD and CAT were significantly decreased in H_2_O_2_-treated cells compared with untreated control cells. The co-treatment of cells with 750 μM H_2_O_2_ and 50 nM VITD resulted in a significant increase in SOD and CAT activities compared to cells exposed to H_2_O_2_ alone (Figure 2B). GSH, an important intracellular antioxidant, can prevent ROS-induced damage. GSH level was significantly decreased in H_2_O_2_-treated cells compared to untreated control cells, while cells co-treated with VITD and H_2_O_2_ showed a significant increase in GSH (compared to cells treated with H_2_O_2_ alone (Figure 2B).

### 3.4. VITD Treatment Modulated the Expression of the Inflammatory Mediators

**Figure 3:**
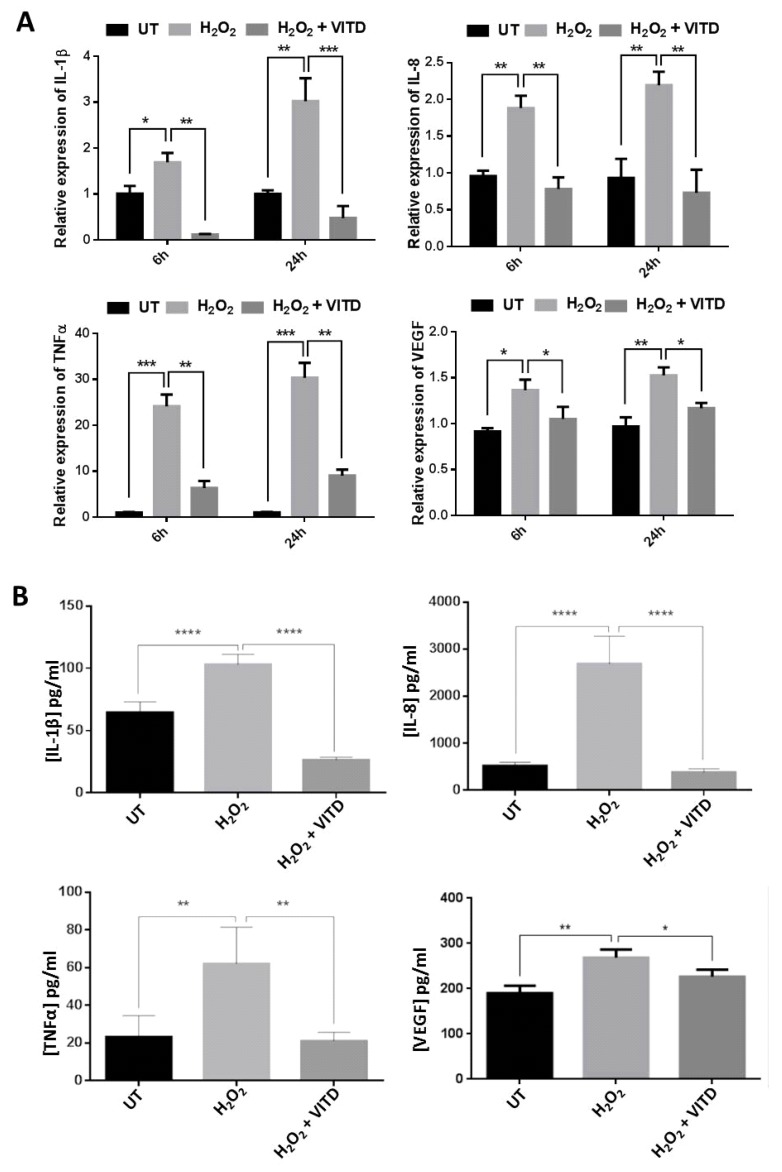
VITD inhibited inflammation. (**A**) ARPE-19 cells were exposed to H_2_O_2_ alone or to H_2_O_2_ + VITD for 6 h or 24 h; the expression of *IL-1β*, *IL-8*, *TNF-α* and *VEGF* in treated and untreated cells was measured by qRT-PCR. (**B**) ARPE-19 cells were exposed to H_2_O_2_ alone or to H_2_O_2_ + VITD for 24 h. The levels of IL-1β, IL-8, TNF-α and VEGF were measured by ELISA. Data are presented as the means ± standard deviation (SD) of three independent experiments. UT: untreated control. * *p* < 0.05, ** *p* < 0.01, *** *p* < 0.001, **** *p* < 0.0001.

Recent studies have suggested that AMD is an inflammatory disease that is associated with increased levels of pro-inflammatory cytokines and the presence of macrophages and other immune cells [1]. VITD treatment has been shown to regulate the gene expression of many inflammatory mediators in various biological systems [32]. In this study, we investigated the expression of *IL-1β*, *IL-8*, *TNF-α* and *VEGFA* genes in ARPE-19 cells treated with 750 μM H_2_O_2_, in cells co-treated with 750 μM H_2_O_2_ and 50 nM VITD, and in untreated control cells. At both 6 and 24 h, the expression levels of *IL-1β*, *TNF-α*, *IL-8*, *IL-18* and *VEGFA* genes were significantly increased in ARPE-19 cells treated with H_2_O_2_ compared to untreated cells; by contrast, at both 6 and 24 h, their expression levels were substantially reduced in ARPE-19 cells co-treated with 750 μM H_2_O_2_ and 50 nM VITD compared to cells treated with 750 μM H_2_O_2_ alone (Figure 3A). We also used ELISA to measure the levels of secreted IL-1β, IL-8, TNF-α and VEGFA in the media of treated and control cells and found that all the examined cytokines were significantly increased in ARPE-19 cells treated with 750 μM H_2_O_2_ when compared to control cells; co-treatment with H_2_O_2_ and VITD markedly decreased the levels of these cytokines when compared to cells exposed to H_2_O_2_ alone (Figure 3B).

### 3.5. VITD Reversed the H_2_O_2_-Induced Change in NRF2 Expression

**Figure 4:**
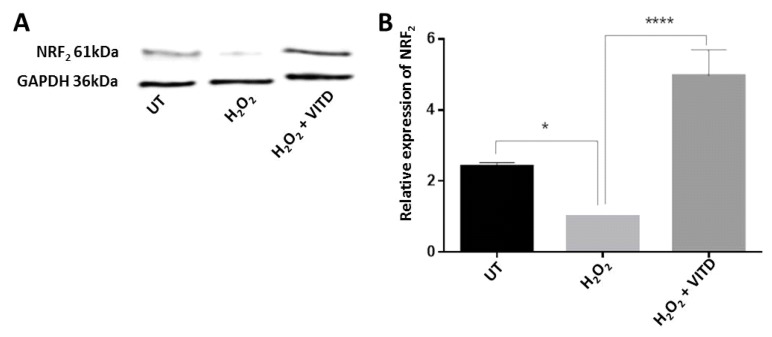
H_2_O_2_ treatment downregulated NRF2 expression, while VITD reversed the effect. (**A**) ARPE-19 cells were treated with H_2_O_2_ alone or H_2_O_2_ + VITD for 24 h; the protein levels of NRF2 in treated and control cells were examined by Western blotting. (**B**) NRF2 protein levels were quantified by normalizing with respect to GAPDH protein. Data are presented as the means ± standard deviation (SD) of three independent experiments. UT: untreated control. * *p* < 0.05, **** *p* < 0.0001.

NRF2 is a redox-sensitive transcription factor that can bind antioxidant elements and activates the expression of antioxidant and detoxifying enzymes. VITD has been reported to control NRF2 expression [32]. In the current study, when ARPE-19 cells were treated with 750 μM H_2_O_2_, NRF2 protein level was significantly decreased compared to untreated control cells; co-treatment with VITD and 750 μM H_2_O_2_ resulted in a marked increase in NRF2 expression when compared to H_2_O_2_ treatment alone (Figure 4).

### 3.6. VITD Regulated IL-33 Expression

**Figure 5:**
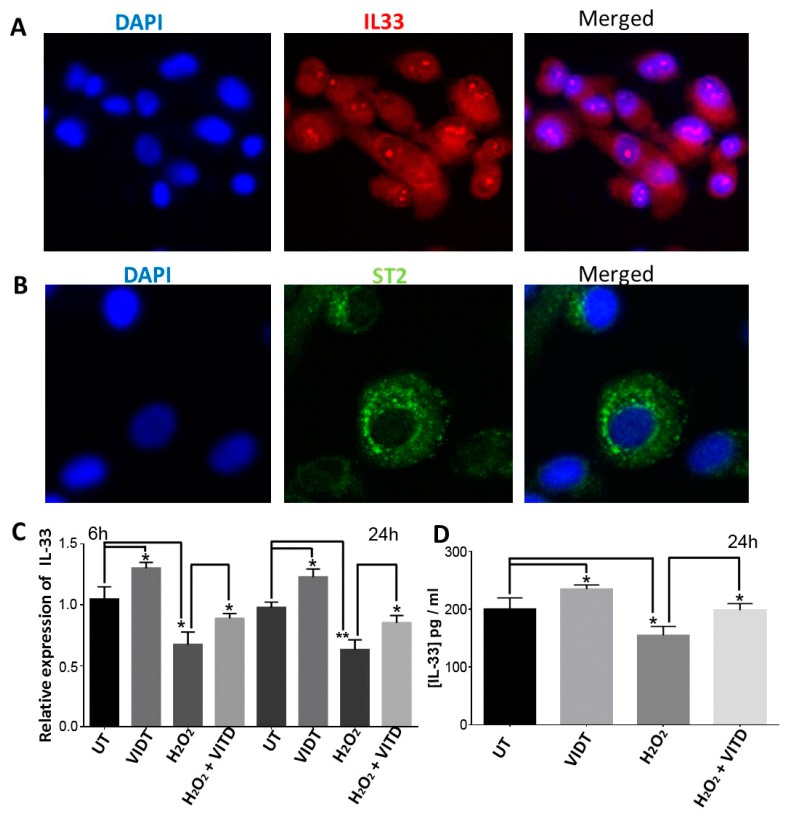
Localization of IL-33 (**A**) and ST-2 (**B**) in ARPE-19 cells using immunostaining with anti-IL-33 antibody and anti-ST2 antibody, respectively (63×). (**C**) Effects of VITD on IL-33 expression in ARPE-19 cells treated with VITD only, H_2_O_2_ alone or H_2_O_2_ + VITD for 6 h or 24 h. IL-33 mRNA levels were quantified by qRT-PCR. (**D**) VITD treatment increased IL-33 protein production. ARPE-19 cells were incubated with VITD only, H_2_O_2_ alone or H_2_O_2_ + VITD for 24 h. Secreted IL-33 protein was measured by ELISA. Data are presented as the means ± standard deviation (SD) of three independent experiments. UT: untreated control. * *p* < 0.05, ** *p* < 0.01.

Previous studies have reported that IL-33 can function as both a proinflammatory and an inflammatory cytokine during the immune response [15], as anti-apoptotic and survival molecules [33] can protect from oxidative stress by increasing SOD activity [34] and can attenuate the development of autoimmune uveitis [35]. IL-33 and its receptor ST2 are abundantly expressed in many tissues such as the central nervous system and the retina [14,35,36]. We used immunostaining to detect IL-33 and ST2 expression in ARPE-19 cells and found that IL-33 was localised in the nucleus (Figure 5A), while ST2 was localised in the cytoplasm, predominantly around the nuclear membrane (Figure 5B). We also measured IL-33 expression in mouse retina and RPE: the expression of IL-33 decreased in the retina and RPE during ageing (Figure S3). We then examined whether H_2_O_2_ and VITD affect IL-33 expression. Using qRT-PCR, we measured the mRNA level of IL-33 in treated and control cells and found that IL-33 expression was significantly decreased in ARPE-19 cells treated with 750 μM of H_2_O_2_ compared to untreated cells, while it was significantly increased in ARPE-19 cells co-treated with 750 μM H_2_O_2_ and 50 nM VITD compared to cells treated with 750 μM H_2_O_2_ alone. VITD treatment alone for 6 and 24 h also significantly increased IL-33 expression when compared to control cells (Figure 5C). We also measured the protein level of IL-33 by ELISA: H_2_O_2_ treatment significantly decreased IL-33 level compared to control cells, while co-treatment with H_2_O_2_ and VITD notably counteracted this change. Exposure to VITD alone resulted in a significantly increased IL-33 level compared to the control cells (Figure 5D).

### 3.7. IL-33 Treatment Upregulated Antioxidant Gene Expression

**Figure 6:**
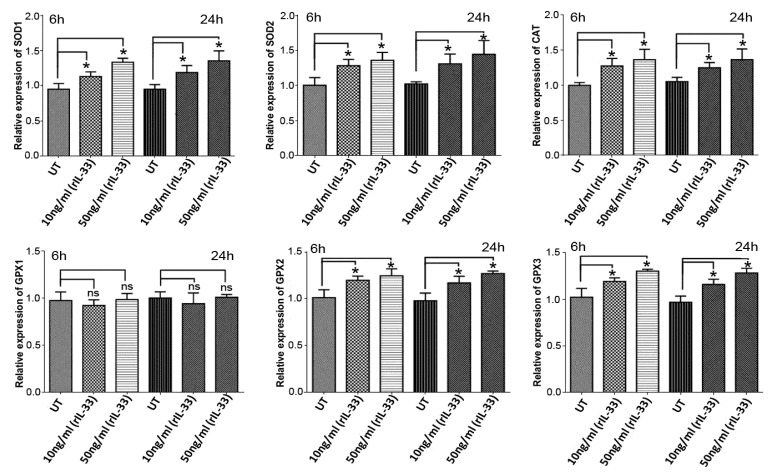
Effects of recombinant human IL-33 on the expression of antioxidant genes. ARPE-19 cells were treated with recombinant human Il-33 (rhIL-33) for 6 or 24 h. The expression of antioxidant genes (*SOD1*, *SOD2*, *CAT*, *GPX1*, *GPX2* and *GPX3*) was measured by qRT-PCR. Data are presented as the means ± standard deviation (SD) of three independent experiments; ns, no significance. * *p* < 0.05.

There is growing evidence that IL-33 can protect cells from apoptosis and oxidative stress and can enhance cell survival in vitro and in vivo [33,37]. The protective function of IL-33 against apoptosis and oxidative stress is believed to occur through its action on the gene expression and production of anti-oxidant scavengers such as superoxide dismutases [34]. In ARPE-19 cells treated with rhIL-33 (10 or 50 ng/mL), the expression of *CAT*, *SOD1*, *SOD2*, *GPX2* and *GPX3* was significantly increased following 6 and 24 h treatments. However, there was no significant change in the expression of *GPX1* following treatment with either 10 or 50 ng/mL rhIL (Figure 6).

## 4. Discussion

It is well established that oxidative stress, inflammation and angiogenesis play a critical role in the development and progression of AMD [38]. VITD has multiple functions including the inhibition of oxidative stress, inflammation, macrophage activation and angiogenesis, thus offering therapeutic potential for AMD. Human RPE cell lines can synthesize active vitamin D (VITD), while VDR and vitamin D synthesis enzymes and metabolism enzymes are also expressed in the neuroretina, RPE and choroid [38]. Early epidemiological studies showed a connection between vitamin D and AMD, while genome-wide association studies demonstrated that the vitamin D metabolism gene (CYP24A1) is a risk factor for AMD [38,39,40]. Lee et al. (2012) reported that Vitamin D treatment resulted in decreased retinal inflammation and Aβ deposition and increased visual function in aged mice [41]. Previously, we reported that VITD can protect cone cells from oxidative damage [17]. Our current data also demonstrate that VITD suppresses oxidative damage and inflammation in RPE cells, further supporting the hypothesis that VITD may play an important role in AMD. 

Although the principal function of the active form of VITD [1,α,25-(OH)2D3] is the regulation of calcium and phosphate homeostasis, it also acts as a steroid hormone, exerting its effect through VDR signalling in target cells. The VDR gene is expressed in a number of ocular structures such as the retina, the cornea and the RPE-choroid and in ocular cell lines such as ARPE-19 and human retinoblastoma (Y79) [25,39], thus demonstrating its regulatory function in the ocular system. VDR is one of the nuclear receptors of steroid hormones that mediate transcriptional processes in a variety of cells. Expression and production of VDR are influenced by factors such as sun exposure, hormones levels and age. VITD itself also regulates VDR expression through two gene expression enhancers containing vitamin D_3_ response element (VDRE): CCAAT/enhancer binding protein-β (C/EBPβ) and runt-related transcription factor-2 (RUNX2) located upstream of the VDR gene’s transcriptional start site (TSS) [42,43]. We also confirmed that VITD stimulated VDR expression in ARPE-19 cells (data not shown) and found that H_2_O_2_ treatment significantly decreased VDR expression, while co-treatment with VITD reversed the H_2_O_2_-caused change (Figure S2).

ROS are highly reactive compounds that cause cellular damage through the oxidation of lipids, proteins and DNA, altering their biochemical characteristics and causing reduced cell viability and subsequent cell death [44]. Many studies have suggested that VITD can improve cell viability and protect against the deleterious effects of ROS in various cell lines [20,22,23]. Similarly, in the current study, a significant reduction of ARPE-19 cell viability was observed in cells treated with H_2_O_2_ at concentrations ranging between 450 and 2000 μM (Figure S1). The reduction in cell viability following H_2_O_2_ treatment was also associated with increased levels of free radicals (ROS) (Figure 1A,B). However, ROS levels were substantially decreased when ARPE-19 cells were treated with a combination of 750 μM of H_2_O_2_ and 50 nM VITD, suggesting that VITD suppressed H_2_O_2_-induced ROS production (Figure 1B). VITD treatment also inhibited lipid peroxidation and reduced cell death (Figure 1C,D). We also found that H_2_O_2_ exposure reduced the expression of antioxidant genes (*SOD1*, *SOD2*, *CAT*, *GPX1*, *GPX2*) and induced the production of inflammatory cytokines (IL-1β, IL-8, TNF-α and VEGF). Co-treatment with VITD counteracted these H_2_O_2_-induced effects (Figure 2; Figure 3). These protective effects of VITD against H_2_O_2_-induced damage can be explained by its effect on the gene and protein expression of many antioxidants via upregulation of different transcription factors, e.g., NRF2, a key transcription factor that modulates the expression of many antioxidants [32]. Our data also demonstrated that H_2_O_2_ caused a significant decrease in NRF2 expression, while co-treatment with VITD markedly increased NRF2 expression (Figure 4). Although VITD plays a critical role in cell proliferation [45] and, as we found, VITD treatment significantly increased cell viability (Figure S1), the current study suggests that the protection of VITD against H_2_O_2_-induced toxicity is through antioxidant signalling pathways.

IL-33 is an important immune mediator in various disorders including neurodegenerative diseases [46]. IL-33 is abundantly expressed in many cell types. Recent studies have reported that IL-33 is expressed in human and mouse RPE and neuroretina [4,35,36]. Our data also demonstrated that both IL-33 and ST2 are expressed in ARPE-19 cells, an observation in accordance with previous reports [4]. Interestingly, IL-33 expression was notably decreased in aged mouse neuroretina and RPE (Figure S3). Liu et al. (2012) reported that amyloid-β1-40 oligomer stimulated IL-33 expression in a human RPE (D407) cell line [47]. However, the data from the present study showed that the mRNA and protein levels of IL-33 were significantly lower in ARPE-19 cells treated with H_2_O_2_, while both IL-33 mRNA expression and protein levels were notably increased following VITD treatment (Figure 5C,D). Increased IL-33 expression has been reported in the vitreous and in retinal Müller cells and mononuclear cells of AMD patients, suggesting that IL-33 may play a pathogenic role in AMD [36]. However, Theodoropoulou et al. (2017) reported that IL-33 regulates tissue remodelling and suppresses murine choroidal neovascularization, a clinical feature of AMD, suggesting that IL-33 may confer some protection in AMD [4]. Additionally, glia-derived IL-33 has been shown to induce myeloid cell infiltration and enhance the recovery of the central nervous system from acute injury [14]. In an experimental autoimmune uveitis mouse model, IL-33 slows down disease progression with decreased retinal inflammation [35]. However, in injured mouse retina, Müller cells have been shown to increase IL-33 production, promoting the release of inflammatory cytokines and chemokines and the recruitment of the myeloid cells, which contribute to photoreceptor cell loss [36].

In the current study, we used co-treatment with VITD and H_2_O_2_ to assess the protection of VITD against H_2_O_2_-induced toxicity. Other researchers have evaluated VITD protection in in vitro cell lines by pre-treatment with VITD [20,23]. Ideally, we would also examine the protection of VITD against H_2_O_2_-induced toxicity by pre-treatment with VITD in ARPE-19 cells. Further preclinical tests in AMD animal models and AMD patient clinical trials are needed to verify the therapeutic potential of VITD for AMD.

## 5. Conclusions

In summary, VITD can possibly protect the retina and RPE from oxidative stress, inflammation and apoptosis through the suppression of pro-inflammatory mediators and by enhancing the antioxidant defence capacity.

## Supplementary Materials

The following are available online at https://www.mdpi.com/2076-3921/8/9/341/s1, Figure S1: Effects of VITD and H_2_O_2_ on cell viability. ARPE-19 cells were exposed to VITD or H_2_O_2_ for 6 hours (**A**) or 24 hours (**B**). Cell viability was measured using MTT assay, Figure S2: (**A**) Expression of vitamin D receptor (VDR) was detected in untreated (UT) and treated (H_2_O_2_ alone or H_2_O_2_ + VITD) ARPE-19 cells by end-point PCR. PCR products were analyzed using agarose gel electrophoresis. (**B**) ARPE-19 cells were exposed to H_2_O_2_ or H_2_O_2_ + VITD for 6 hours or 24 hours; expression of VDR was measured by qRT-PCR. Figure S3: Expression of IL-33 in the retinas (**A**) and RPE (**B**) of mice at three age points was measured by qRT-PCR.
