# Supplementary material for: Vitamin D Attenuates Oxidative Damage and Inflammation in Retinal Pigment Epithelial Cells
Source: Antioxidants (Basel). 2019 Aug 24;8(9):341. doi: 10.3390/antiox8090341 (PMC6770403; doi:10.3390/antiox8090341)
Supplement: Supplementary file 1 [file antioxidants-08-00341-s001.pdf]

Supplementary Data

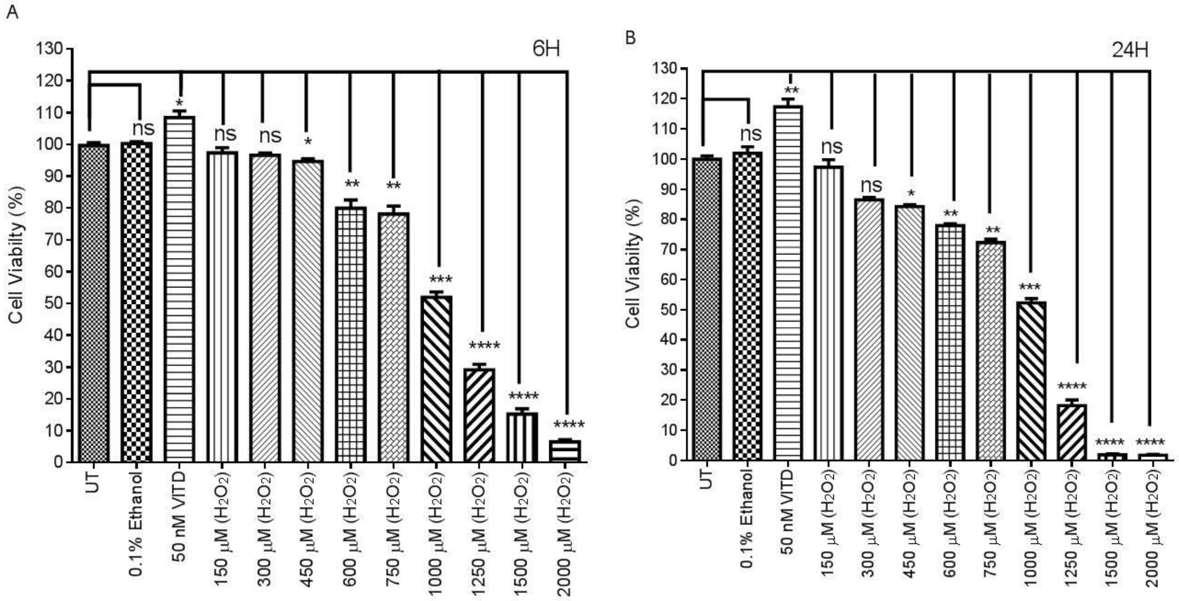

Figure S1 Effects of VITD and H_2O_2 on cell viability. ARPE-19 cells were exposed to VITD or H_2O_2 for 6 hours (A) or 24 hours (B). Cell viability was measured using MTT assay. Data are presented as the means± standard error (SD) of three independent experiments. ns, no significance. *p<0.05, **p<0.01, ***p<0.001, ****p<0.0001.

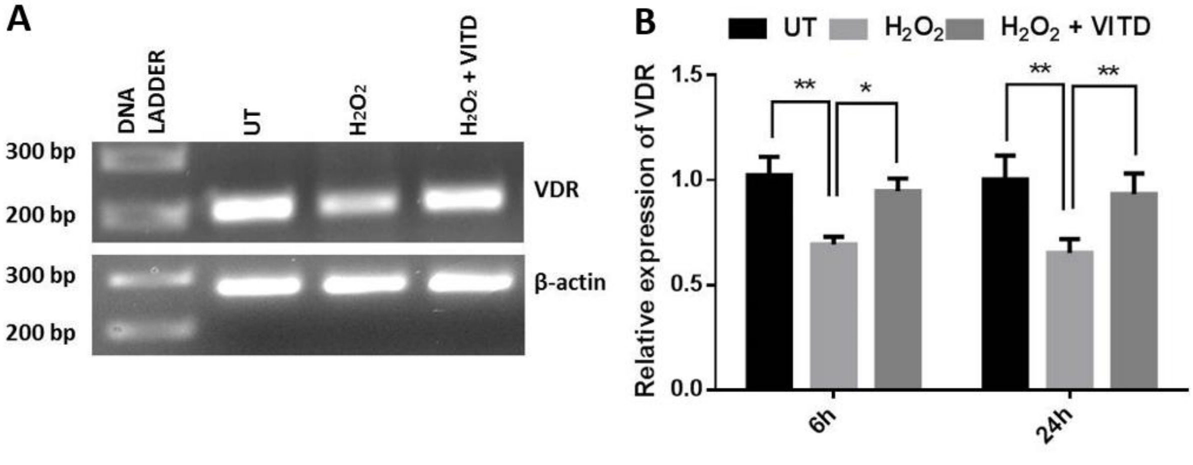

Figure S2 (A) Expression of vitamin D receptor (VDR) was detected in untreated (UT) and treated (H_2O_2 alone or H_2O_2+VITD) ARPE-19 cells by end-point PCR. PCR products were analyzed using agarose gel electrophoresis. (B) ARPE-19 cells were exposed to H_2O_2 or H_2O_2+VITD for 6 hours or 24 hours; expression of VDR was measured by qRT-PCR. Data

are presented as the means± standard error (SD) of three independent experiments. *p<0.05, **p<0.01.

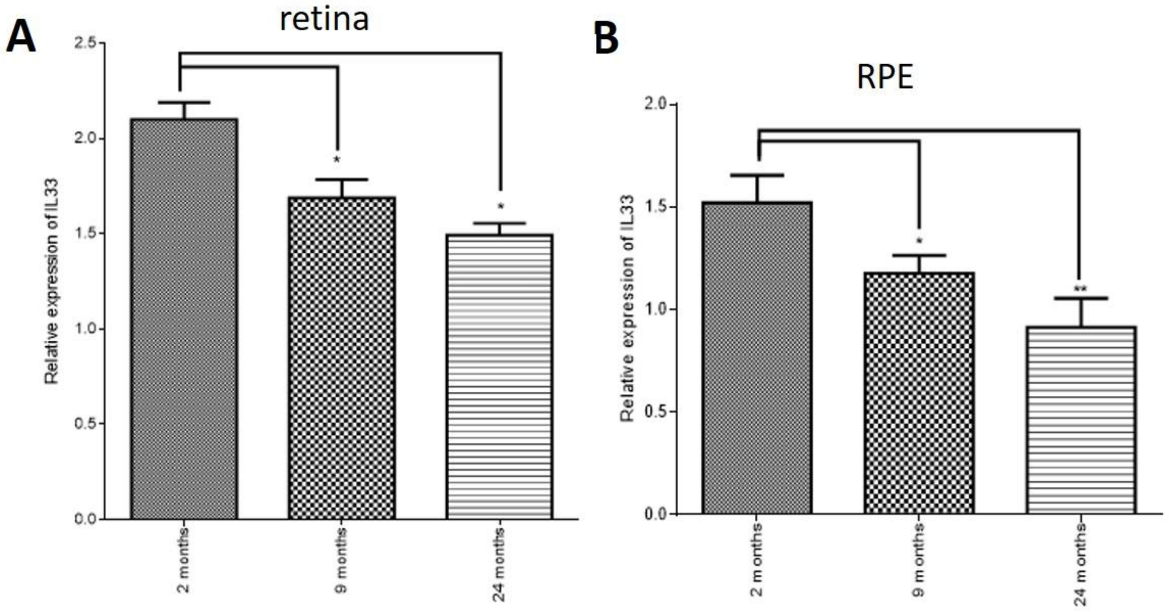

Figure S3 Expression of IL-33 in the retinas (A) and RPE (B) of mice at three age points was measured by qRT-PCR. Data are presented as the means± standard error (SD) of three independent experiments. UT: untreated control. *p<0.05, **p<0.01.
